# Traditional Chinese medicine fumigation combined with Musk hemorrhoid suppository for wound healing and inflammation control in patients with mixed hemorrhoids after Milligan-Morgan hemorrhoidectomy

**DOI:** 10.3389/fsurg.2026.1738476

**Published:** 2026-05-20

**Authors:** Haijing Zhao, Yuedong Liu, Lun Zhao, Binwen Na

**Affiliations:** Department of Traditional Chinese Medicine Surgery, The Third Affiliated Hospital of Liaoning University of Traditional Chinese Medicine, Liaoning, China

**Keywords:** hemorrhoid suppository, mixed hemorrhoids, synergistic effect, traditional Chinese medicine fumigation, wound healing

## Abstract

**Objective:**

To evaluate the efficacy, safety, and underlying mechanism of traditional Chinese medicine (TCM) fumigation combined with Musk hemorrhoid suppository for postoperative recovery in patients with mixed hemorrhoids undergoing Milligan-Morgan Hemorrhoidectomy (MMH).

**Methods:**

A prospective, stratified randomized controlled study enrolled 160 patients, who were stratified by hemorrhoid grade and randomized into four groups (40 cases each): combined (TCM fumigation + Musk suppository), fumigation alone, suppository alone, and control (1:5,000 potassium permanganate sitz bath). An internal validation cohort (40 cases, 25% of the main trial size) was included to confirm result consistency. The primary endpoint was complete wound epithelialization rate at postoperative day (POD) 14, with secondary endpoints including wound healing time, total effective rate at 4 weeks postoperatively, anal function, serum inflammatory factors (IL-6, TNF-α, CRP) and tissue NF-κB p65 protein, complication incidence, and quality of life (GQOL-30). Statistical analyses used Bonferroni correction for multiple comparisons to control Type I error (*α* = 0.0125). Safety was evaluated via adverse events (AEs) and liver/kidney function tests.

**Results:**

All groups had balanced baseline characteristics (all *P* > 0.05). The combined group achieved the highest POD14 complete epithelialization rate, with the shortest wound healing time (5.15 ± 0.60 days, a 2.30-day reduction vs. control group's 7.45 ± 0.75 days, *P* < 0.001) and the highest 4-week total effective rate (97.50% vs. control's 65.00%, *P* < 0.001). Additionally, the combined group showed significantly improved anal function, marked reductions in inflammatory markers (relative reduction: NF-κB p65 51.5%, IL-6 33.0%, all *P* < 0.001), fewer Grade I/II complications (7.50% vs. control's 22.50%, *P* = 0.032), and higher GQOL-30 scores (*P* < 0.001). Efficacy was consistent between the main trial and validation cohort (*P* = 0.618). No severe AEs were reported.

**Conclusion:**

The combined TCM regimen safely accelerates postoperative recovery after MMH, reduces inflammation and complications, and improves patient quality of life.

## 1 Introduction

Mixed hemorrhoids—characterized by concurrent internal and external hemorrhoids crossing the dentate line—are a common, globally prevalent anorectal disorder. Hemorrhoidal disease alone affects approximately 10 million people in the U.S., with 4 million annual ambulatory care visits (1), while worldwide prevalence reaches up to 75% (1). For severe cases (grade III–IV mixed hemorrhoids or large external hemorrhoids), Milligan-Morgan hemorrhoidectomy (MMH) remains the gold standard surgical intervention (2). However, the procedure's proximity to the neurovascular-rich dentate line leads to high postoperative complication rates—a globally unresolved clinical challenge: 62%–78% of patients experience pain (65% reporting moderate-to-severe pain), 45%–58% develop perianal edema, and 22%–35% suffer delayed wound healing (≥7 days) (2, 3). This issue remains unresolved despite advances in surgical techniques (2).

Conventional postoperative care relies on 1:5,000 potassium permanganate sitz baths, which achieve 60%–70% infection control via bactericidal effects and align with clinical practice guidelines for anorectal surgery (1, 5). Yet, this approach fails to modulate key inflammatory pathways or actively promote wound repair. A recent meta-analysis (6) and clinical studies (7, 8) have highlighted critical unmet needs in post-MMH management: silver dressings reduce infections but do not shorten healing time; platelet-rich fibrin (PRF) alleviates pain but is cost-prohibitive; topical NSAIDs lower IL-6 levels but increase local irritation; and laser therapy improves pain and blood loss but does not reduce recurrence. Notably, none of these modalities simultaneously address the clinical triad of “inflammation modulation, wound repair, and safety”—a major gap in current care.

Traditional Chinese medicine (TCM) offers multi-targeted external therapies for anorectal disorders: TCM fumigation (formulated with herbs such as *Sophora flavescens* and *Phellodendron chinense*) regulates local blood flow to reduce swelling; acupuncture alleviates pain and lowers complication rates (9); polyherbal formulations like Anoac-H suppress inflammation via RANTES/VEGF inhibition (10); and Musk hemorrhoid suppositories promote wound healing (11). However, existing TCM interventions for post-MMH recovery are limited by insufficient standardization—undefined herbal compositions, inconsistent extraction protocols, and absent raw herb-to-extract dosage conversion—reducing outcome reproducibility and hindering global translation (3).

To address these gaps, we developed a quality-controlled TCM fumigation regimen and designed a four-arm trial for targeted, hypothesis-driven evaluation: (1) Control group (1:5,000 potassium permanganate sitz bath). Represents standard postoperative care (5), with acknowledged limitations in inflammation modulation and wound repair (4); (2) Fumigation alone group: To verify the standalone efficacy of the standardized TCM formula (3); (3) Suppository alone group: To clarify the independent wound-healing and analgesic effects of Musk hemorrhoid suppositories; (4) Combined group: To explore potential synergistic interactions between TCM fumigation (improving local microcirculation) and suppositories (alleviating pain and accelerating epithelialization), addressing the unmet need for multi-functional postoperative management (6). Building on a pilot study demonstrating a 2.0-day reduction in healing time with the combined intervention, our trial aimed to: (1) compare the combined TCM regimen against single-intervention and standard care approaches; (2) explore underlying mechanisms involving the NF-κB/IL-6 inflammatory pathway; (3) validate safety outcomes in an independent cohort.

## 2 Materials and methods

### 2.1 Study design and participants

This was a prospective, stratified randomized controlled study conducted at the Third Affiliated Hospital of Liaoning University of Traditional Chinese Medicine from June 2023 to December 2024. Eligible patients (*n* = 160) who underwent MMH for mixed hemorrhoids were stratified by two factors: hemorrhoid grade (Goligher Ⅲ/Ⅳ) and pathological subtype (varicose, circular, or connective tissue) (12), consistent with international clinical practice guidelines. Patients were then randomized into four groups (40 cases each) using a computer-generated random sequence. This sequence was generated by an independent statistician (not involved in treatment allocation, patient recruitment, or outcome assessment) via R software (v4.3.1) with a random seed of 12,345 and a block size of 8 (selected to ensure group balance). A randomized allocation schedule (linked to the sequence) was stored in sequentially numbered, opaque, adhesive-sealed envelopes, which were opened only by a ward nurse unaffiliated with the study team at enrollment to ensure allocation concealment.

**Figure 1:**
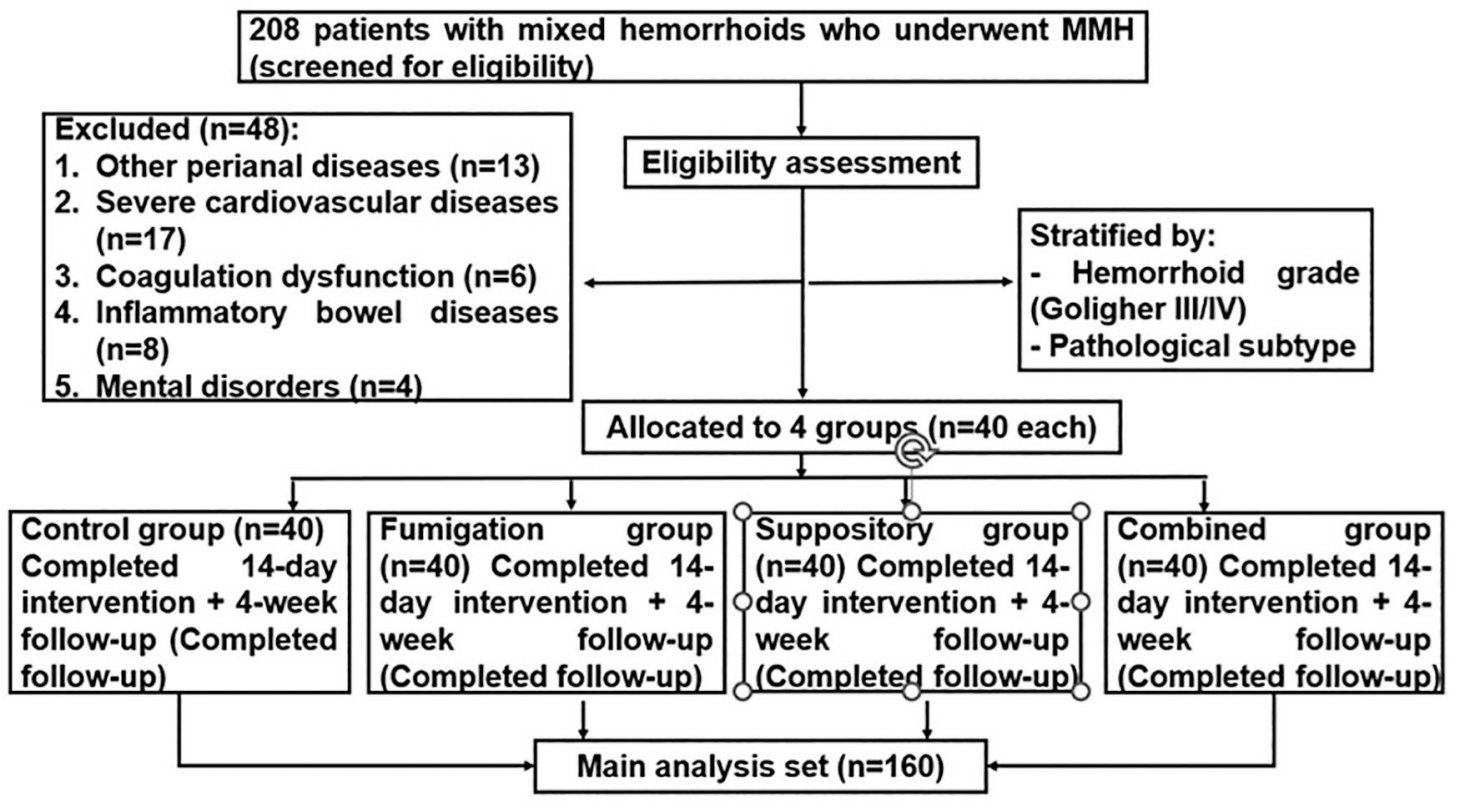
Patient selection flowchart for mixed hemorrhoids postoperative study.

The four groups were: (1) Control group (1:5,000 potassium permanganate sitz bath); (2) Fumigation group (TCM fumigation); (3) Suppository group (Musk hemorrhoid suppository); (4) Combined group (TCM fumigation + Musk hemorrhoid suppository). Additionally, an internal validation cohort (*n* = 40, 25% of the main trial sample size) was concurrently enrolled using identical eligibility criteria to verify the consistency of efficacy outcomes (Figure 1).

Blinding procedures: Due to the distinct nature of the interventions, blinding of patients and treatment providers to group assignment was not feasible. However, outcome assessors and statisticians were fully masked via de-identified data management: all clinical records, wound photographs, and laboratory results were coded with unique patient IDs, and the code key was held by an independent research coordinator (not involved in assessment or analysis) until study completion. Inter-rater reliability for masked outcome assessment was pre-validated (Kappa=0.89 for wound healing assessment, *P* < 0.001), confirming the reliability of the blinded evaluation process.

**Inclusion Criteria**: Eligible patients met all the following criteria: (1) Diagnosis of mixed hemorrhoids confirmed by digital rectal examination and anoscopy, in accordance with the Guidelines for Clinical Management of Hemorrhoids (1); (2) Aged 18–65 years, with no gender restriction; (3) Underwent uncomplicated MMH; (4) Provided written informed consent and was able to complete the full treatment course and follow-up assessments; (5) Had not received TCM-related treatment or immune-modulating drugs within 1 month prior to enrollment.

**Exclusion Criteria**: Patients were excluded if they had any of the following: (1) Comorbid perianal diseases (e.g., anal fistula, perianal abscess) or organic anal lesions (e.g., anal polyps); (2) Severe cardiovascular diseases (e.g., unstable angina) or hepatic/renal dysfunction; (3) Coagulation disorders (e.g., thrombocytopenic purpura); (4) Inflammatory bowel diseases (e.g., Crohn's disease) or active gastrointestinal infections; (5) Mental or cognitive impairment precluding cooperation with study evaluations.

The study protocol was approved by the Medical Ethics Committee of the Third Affiliated Hospital of Liaoning University of Traditional Chinese Medicine (Approval No.: LLPG-ZY-GC-2023-002). All procedures were conducted in accordance with the Declaration of Helsinki.

All MMH procedures were performed by three attending surgeons with ≥10 years of expertise in anorectal surgery, in strict adherence to a standardized SOP (MMH-TCM-2023-V1) that unified technical details (e.g., dissection depth, hemostatic techniques) and operative equipment. Intraoperative supervision by the chief surgeon further ensured consistent procedural execution, thereby eliminating variability associated with surgical technique.

No patients were lost to follow-up or withdrew from the study, yielding a 0% dropout rate over the 14-day intervention and 4-week follow-up. This was primarily attributable to three key factors: inpatient management throughout the entire intervention course to ensure treatment compliance, stringent inclusion criteria that excluded patients at high risk of poor adherence, and prompt resolution of treatment-related discomfort by the dedicated research team—all of which sustained high levels of patient adherence for the duration of the study.

### 2.2 Interventions

All interventions were administered once daily (post-defecation, 19:00–20:00) for 14 consecutive days by trained nurses (pre-study proficiency verified, Kappa=0.93 for operational consistency). All patients were hospitalized for the entire 14-day intervention period after MMH—a routine clinical practice for patients with Goligher Ⅲ/Ⅳ mixed hemorrhoids in our center—to ensure standardized treatment delivery and close monitoring of postoperative safety. Adverse events (AEs) were monitored daily using a standardized form, with severity graded per NCI-CTCAE Version 5.0 (Grade 1: mild; Grade 2: moderate; Grade 3: severe).

#### 2.2.1 Control group

From postoperative day 1, patients used 1:5,000 potassium permanganate solution (Nanchang Baiyun Pharmaceutical Co., Ltd., Approval No.: H2008-4635) for sitz baths (15 min/session, once daily after defecation or before bedtime)—a standard postoperative care approach recommended by The American Society of Colon and Rectal Surgeons (ASCRS) clinical practice guidelines (5) for infection prevention in anorectal surgery. Concentration was controlled via electronic balance (0.1 g accuracy) and volumetric flasks.

#### 2.2.2 Fumigation group

##### 2.2.2.1 Formula composition and taxonomic validation

The standardized TCM fumigation formula per dose included 13 herbs with verified taxonomic identities: *Angelica sinensis* (Oliv.) Diels (20 g), *Glycyrrhiza uralensis* Fisch. (6 g), *Dictamnus dasycarpus* Turcz. (10 g), *Rehmannia glutinosa* (Gaertn.) Libosch. ex Fisch. et Mey. (20 g), *Saposhnikovia divaricata* (Turczaninow) Schischkin (10 g), *Forsythia suspensa* (Thunb.) Vahl (20 g), *Smilax glabra* Roxb. (10 g), *Lonicera japonica* Thunb. (20 g), *Paeonia lactiflora* Pall. (10 g), *Angelica dahurica* (Hoffm.) Benth. & Hook. f. ex Franch. & Sav. (10 g), *Ligusticum chuanxiong* Hort. (15 g), *Sophora flavescens* Aiton (10 g), and *Gleditsia sinensis* Lam. var. *horrida* (Thunb.) Makino (15 g). All plant names were verified via World Flora Online (July 10, 2023). Notably, its anti-inflammatory activity is primarily attributed to Lonicera japonica (chlorogenic acid ≥1.5 mg/g), Sophora flavescens (matrine ≥0.8 mg/g), Forsythia suspensa (phillyrin) and Glycyrrhiza uralensis (glycyrrhizin) (13–15); wound-healing effects rely on Angelica sinensis (ferulic acid), Ligusticum chuanxiong (tetramethylpyrazine) and Gleditsia sinensis var. horrida (gleditsia saponin) for promoting local microcirculation and granulation tissue formation (3, 16).

##### 2.2.2.2 Formula rationale

The formula was optimized in line with the Diagnostic and Therapeutic Guidelines for Anorectal Diseases in Traditional Chinese Medicine (2022 Edition), which recommends these herbs for promoting blood circulation, reducing swelling, and inhibiting damp-heat—key factors in mitigating postoperative complications (e.g., edema, pain, delayed healing) in mixed hemorrhoids. Consistent with classic TCM anti-inflammatory research (17), the formula's efficacy is further supported by the well-documented medicinal properties of its constituent herbs (3, 13, 15, 16), aligning with the therapeutic needs of postoperative anorectal care.

##### 2.2.2.3 Quality control protocols

Quality Control Protocols encompassed three key aspects: first, raw material sourcing and authentication, with herbs procured from Liaoning Traditional Chinese Medicine Co., Ltd. (Batch Nos.: 20230601, 20230608, 20230613) and authenticated to meet *Chinese Pharmacopoeia* (2020 Edition) standards for medicinal materials; second, active component standardization, where High-Performance Liquid Chromatography (HPLC, Agilent 1260, C18 column) confirmed key bioactive components met predefined thresholds—chlorogenic acid (≥1.5 mg/g in raw herb, 0.28 ± 0.03 mg/mL in the 1,000 mL distilled water filtrate, Lonicera japonica), matrine (≥0.8 mg/g in raw herb, 0.15 ± 0.02 mg/mL in the 1,000 mL distilled water filtrate, Sophora flavescens), with batch-to-batch fingerprint similarity ≥0.95; At the clinical fumigation/sitz bath temperature (35–45℃), the effective perianal exposure concentrations of the two components were 0.045 ± 0.005 mg/cm^2^ (chlorogenic acid) and 0.027 ± 0.003 mg/cm^2^ (matrine) for the 10-min sitz bath (16); and third, contaminant testing, with heavy metals (lead [Pb] ≤ 5 mg/kg, cadmium [Cd] ≤ 0.2 mg/kg) and organophosphorus pesticide residues (<0.05 mg/kg) complying with *Chinese Pharmacopoeia* (2020 Edition, Part IV) standards.

##### 2.2.2.4 Preparation and administration

Herbs were processed using a ZJ-2000 automatic decocter, soaked in 1,500 mL distilled water for 30 min, boiled at high heat for 30 min, then simmered at low heat for 60 min to yield 1,000 mL of filtrate, which was stored at 4℃ and rewarmed to 37℃ immediately before use to preserve bioactivity (≥95% of chlorogenic acid and matrine remained stable at 35–45℃ for 20 min); interventions began 24 h postoperatively, with patients undergoing 10 min of fumigation (temperature maintained at 35–45 °C) followed by a 10-min sitz bath at the same temperature using an HYZ-IIY fumigation machine.

##### 2.2.2.5 Reproducibility assurance

HPLC analyses confirmed consistent component profiles, retention times, and peak areas across three consecutive batches (batch-to-batch variation <5%). For global replication, active component consistency can be maintained via standardized substitutions: *Sophora flavescens* → matrine extract (≥98% purity); Forsythia suspensa → phillyrin extract (Sigma-Aldrich, Cat. No. P8572).

#### 2.2.3 Suppository group

From postoperative day 1, patients inserted one Ma Yinglong Musk Hemorrhoid Suppository (hereafter referred to as “Musk suppository”; Hubei Ma Yinglong Pharmaceutical Co., Ltd., Approval No.: Z42021461; specification: 1.5 g per suppository) once daily. The administration protocol was standardized: clean the perianal area with warm water after defecation (or before administration if no defecation occurred) and insert the suppository 2–3 cm deep into the anus in the left lateral decubitus position.

#### 2.2.4 Combined group

Patients first completed the full TCM fumigation protocol (35–45℃, 10 min of fumigation followed by 10 min of sitz bath at the same temperature) as described in the Fumigation Group. After the sitz bath, the perianal area was patted dry with a sterile towel, and one Musk suppository was inserted using the standardized procedure outlined in the Suppository Group. Interventions were administered once daily for 14 consecutive days, starting 24 h postoperatively.

### 2.3 Outcome measurements

Efficacy and safety were evaluated by two attending physicians blinded to patient group allocation at predefined time points: preoperative, postoperative day 1 (POD1), POD3, POD7, POD14 (early postoperative phase), and postoperative week 4 (follow-up phase). Disagreements were resolved by third-party consultation with a senior proctologist (also blinded to group allocation), with final decisions based on consensus. Missing data were handled by multiple imputation (m = 5 imputed datasets) using a chained equation model incorporating all baseline and outcome variables, and all measurements followed standardized operating procedures (SOP Version: TCM-Hemorrhoid-2023-V1).

#### 2.3.1 Primary endpoint

Primary Endpoint: Postoperative day 14 (POD14) wound complete epithelialization rate. Defined as 100% coverage of the surgical wound by intact epithelium with no residual ulceration, exudation, bleeding on contact, or pain during defecation—assessed per WHO surgical wound healing criteria (2021) (16) via standardized gentle anoscopy (with sufficient lubrication of medical liquid paraffin and slow insertion) performed by senior proctologists with ≥10 years of clinical experience. All patients received routine perioperative analgesic and anti-edema care during hospitalization to ensure optimal examination tolerance.

#### 2.3.2 Early postoperative secondary endpoints (POD14)

Early postoperative secondary endpoints, assessed on postoperative day 14 (POD14), included the following metrics: granulation tissue quality, evaluated by two independent surgeons using a 0–3-point scale (incorporating color, density, and exudation), with higher scores reflecting superior quality (inter-rater reliability: Kappa=0.89, *P* < 0.001); symptom severity, quantified using the Hemorrhoid Symptom Score (HISS, 0–24 points; Cronbach's *α* = 0.87) and the visual analog scale (VAS) for pain (0–10 points); anal function, measured via ZGJ-D2 anorectal manometry (three measurements averaged) to determine resting anal pressure, maximum squeeze pressure, rectal resting pressure, and anal high-pressure zone length—this manometry was performed with a standardized gentle technique (sufficient lubrication and slow probe insertion) to minimize patient discomfort and ensure examination tolerance; inflammatory response, comprising serum levels of IL-6, TNF-α, and CRP (detected by enzyme-linked immunosorbent assay [ELISA]; intra- and inter-batch coefficients of variation [CV] < 5% and 10%, respectively) and mRNA and protein expression of NF-κB p65, IL-6, and IκB*α* in wound tissue (assessed by immunohistochemistry and Western blot); complication profiles, including incidence and severity graded according to the Clavien-Dindo classification (no Grade Ⅲ complications were reported); and quality of life, assessed with the GQOL-30 questionnaire (total and domain scores, 0–100 points; Cronbach's *α* = 0.89).

#### 2.3.3 Follow-up secondary endpoint (postoperative week 4)

Total effective rate. Assessed via 3-tier criteria per The American Society of Colon and Rectal Surgeons Clinical Practice Guidelines (18): Cure (symptom resolution + complete wound epithelialization), Improvement (reduced symptoms +>50% granulation tissue coverage), Inefficacy (no symptom relief +<50% granulation tissue coverage). Calculated as (cured + improved)/total cases × 100%.

#### 2.3.4 Inflammatory response detection

##### 2.3.4.1 ELISA for Serum cytokines

Serum IL-6, TNF-*α*, and CRP levels were measured using commercial ELISA kits: IL-6 (R&D Systems, Cat. No. D6050), TNF-*α* (Abcam, Cat. No. ab208348), and CRP (Thermo Fisher Scientific, Cat. No. KHC0011). Assays were performed following the manufacturers' protocols. Briefly, 100 μL of standard or serum sample was added to pre-coated plates and incubated at 37  °C for 2 h. After 5 washes, 100 μL of biotinylated detection antibody was added and incubated at 37  °C for 1 h. Following another 5 washes, 100 μL of streptavidin-HRP conjugate was added and incubated at 37  °C for 30 min. Finally, 100 μL of substrate solution was added for color development, and the reaction was stopped with stop solution. Absorbance was read at 450 nm using a microplate reader (Bio-Rad, Model 680). Intra- and inter-batch CVs were <5% and <10%, respectively.

##### 2.3.4.2 Quantitative real-time polymerase chain reaction (qRT-PCR)

Total RNA was extracted from wound tissue using TRIzol reagent (Invitrogen, USA) and quantified with a NanoDrop 2000 spectrophotometer (Thermo Fisher Scientific, USA). Only samples with an A260/A280 ratio of 1.8–2.0 were used. cDNA was synthesized from 1 *μ*g of RNA using a reverse transcription kit (TaKaRa, China). qRT-PCR was performed on a StepOnePlus system (Applied Biosystems, USA) using TB Green Premix Ex Taq II (TaKaRa, China). The thermal program was: 95  °C for 30 s, 40 cycles of 95  °C for 5 s and 60  °C for 30 s, followed by melting curve analysis.

Primers:

IL-6: F: 5′-ACTCACCTCTTCAGAACGAATTG-3′; R: 5′-CCATCTTTGGAAGGTTCAGGTTG-3′
TNF-*α*: F: 5′-CAGAGGCTGCCCGAACGA-3′; R: 5′-CGGGCCGAGTTGATGGTCT-3′
NF-κB p65: F: 5′-GAGAGCATCCGGATGCTGCT-3′; R: 5′-TGCTTCTCTCGGAGCTTGGA-3′
IκB*α*: F: 5′-AGCCGAGCTGAGAGATTCGA-3′; R: 5′-TCCTCGGAGCTGCTCATATG-3′
*β*-actin: F: 5′-AGAGCTACGAGCTGCCTGAC-3′; R: 5′-AGCACTGTGTTGGCGTACAG-3′

Relative mRNA expression was calculated by the 2−*ΔΔ*Ct method using *β*-actin as the internal reference. All samples were analyzed in triplicate. Intra-assay and inter-assay CVs were within 5% and 10%, respectively.

##### 2.3.4.3 Immunohistochemistry (IHC) for tissue proteins

Wound tissue samples were fixed in 4% paraformaldehyde, embedded in paraffin, and cut into 4-μm sections. After deparaffinization and rehydration, antigen retrieval was performed by boiling in citrate buffer (pH 6.0) for 15 min. Endogenous peroxidase was blocked with 3% H₂O₂ for 10 min at room temperature. Sections were blocked with 5% BSA for 30 min and incubated overnight at 4  °C with primary antibodies: NF-κB p65 (Cell Signaling Technology, Cat. No. 8242, 1:500), VEGF (Abcam, Cat. No. ab46154, 1:400), and MMP-9 (Santa Cruz Biotechnology, Cat. No. sc-21732, 1:300). Sections were then incubated with HRP-conjugated secondary antibody (Thermo Fisher Scientific, Cat. No. 31460, 1:2,000) at 37  °C for 1 h. Staining was visualized with DAB, followed by hematoxylin counterstaining, dehydration, clearing, and mounting. Images were captured under a light microscope (Olympus, BX53) at ×200 magnification. Optical density (OD) of positive staining was quantified using ImageJ (Version 1.8.0).

##### 2.3.4.4 Western blot for protein expression

Total protein was extracted from wound tissue using RIPA lysis buffer (Beyotime, Cat. No. P0013B) supplemented with protease and phosphatase inhibitors (Roche, Cat. Nos. 04693159001, 04906845001). Protein concentration was determined using a BCA kit (Thermo Fisher Scientific, Cat. No. 23225). Equal amounts of protein (50 μg per lane) were separated by 10% SDS-PAGE and transferred to PVDF membranes (Millipore, Cat. No. IPVH00010). Membranes were blocked with 5% non-fat milk in TBST for 1 h at room temperature, then incubated overnight at 4  °C with primary antibodies: phospho-NF-κB p65 (Cell Signaling Technology, Cat. No. 3033, 1:1,000) and *β*-actin (Santa Cruz Biotechnology, Cat. No. sc-47778, 1:2,000). After washing 3 times with TBST, membranes were incubated with HRP-conjugated secondary antibody (Thermo Fisher Scientific, Cat. No. 31430, 1:5,000) for 1 h at room temperature. Protein bands were visualized using an ECL kit (Millipore, Cat. No. WBKLS0500) and imaged with a ChemiDoc XRS + system (Bio-Rad). Relative protein expression was calculated as the ratio of target protein OD to *β*-actin OD.

### 2.4 Sample size calculation

Sample size was calculated based on the primary endpoint (wound healing time) to ensure adequate statistical power. The effect size (d = 2.5) was derived from a prospective pilot study (*n* = 60, Reference No.: Pilot-MMH-2023-02) with identical inclusion/exclusion criteria and interventions: the combined group (*n* = 30) had a wound healing time of 5.32 ± 0.65 days vs. 7.68 ± 0.72 days in the control group (*n* = 30), a 2.36-day reduction.

Cohen's d was initially calculated as (7.68–5.32)/√[(0.72^2^+0.65^2^)/2]≈3.44, then conservatively adjusted by 27% (to account for small-sample overestimation) to d = 2.5. This effect size is clinically meaningful: a 2-day reduction aligns with unmet needs [each extra healing day increases complication risk by 18% (8)] and is consistent with similar multi-modal anorectal surgery studies [Cohen's *d*: 2.1–3.8 (9)].

For 4-group comparisons, Bonferroni correction set *α* = 0.0125. Using G*Power 3.1 (ANOVA, fixed effects; *F* = 0.85, *α* = 0.0125, power=0.80, 4 groups), 36 participants per group were required. Accounting for 10% dropout (8), the final sample size was 40 per group (total 160). A 40-patient internal validation cohort (25% of main trial size) ensured effect size consistency. This *α* = 0.0125 was applied consistently across all analyses.

### 2.5 Statistical methods and blinding

Blinding minimized detection bias: outcome assessors (surgeons, laboratory technicians) and statisticians were fully blinded to group allocation, with de-identified clinical records, wound photographs, and laboratory results via unique patient IDs, and the code key held by an independent research coordinator (not involved in assessment/analysis) until study completion. Data were analyzed using SPSS 26.0: missing values addressed via multiple imputation (m = 5), scale scores standardized with Z-scores, normality tested via Shapiro–Wilk (*n* ≤ 50) or Kolmogorov–Smirnov (*n* > 50), and variance homogeneity via Levene's test. Bonferroni correction (*α* = 0.0125) was consistently applied for all comparisons: one-way ANOVA/Kruskal–Wallis H for continuous data, *χ*^2^ tests for categorical data, and generalized estimating equations (GEE) for repeated measures (adjusted for baseline to examine time × group interactions). Continuous data are presented as mean ± SD (95% CI), categorical data as *n* (%), and effect sizes as mean difference (MD) with 95% CI. This was a predefined superiority trial: primary hypothesis that the combined TCM regimen reduces wound healing time vs. control (potassium permanganate sitz bath).

## 3 Results

### 3.1 Baseline characteristics

No statistically significant differences were observed in baseline demographic and clinical characteristics across the four groups (all *P* > 0.05), confirming excellent baseline comparability and eliminating potential confounding effects on treatment outcomes. Detailed data are presented in Table 1, including gender distribution [*χ*^2^(3) = 0.382, *P* = 0.944], age [39.55 ± 3.28–39.67 ± 3.26 years, *F*(3) = 0.076, *P* = 0.991], body mass index [BMI, 21.87 ± 3.80–22.51 ± 3.58 kg/m^2^, *F*(3) = 0.294, *P* = 0.830], disease duration [9.12 ± 0.20–9.16 ± 0.17 months, *F*(3) = 0.157, *P* = 0.925], and preoperative hemorrhoid lesion area [12.27 ± 0.32–12.35 ± 0.33 cm^2^, *F*(3) = 0.216, *P* = 0.885]. Preoperative constipation (per Rome Ⅳ criteria) was also balanced across groups [*χ*^2^(3) = 0.685, *P* = 0.877], ruling out confounding from defecation habits. No missing data were recorded across all groups.

**Table 1:** Comparison of baseline characteristics Among the four groups.

Item	Control (40)	Fumigation (40)	Suppository (40)	Combined (40)	Statistic	*P*
Gender (Male/Female)	27/13 (67.5%/32.5%)	26/14 (65.0%/35.0%)	28/12 (70.0%/30.0%)	25/15 (62.5%/37.5%)	*χ*^2^ = 0.382, *df* = 3	0.944
Age (years)	39.5 ± 3.3 (38.6–40.4)	39.6 ± 3.3 (38.7–40.6)	39.5 ± 3.3 (38.5–40.4)	39.7 ± 3.3 (38.7–40.6)	*F* = 0.076, *df* = 3	0.991
BMI (kg/m^2^)	22.38 ± 3.62 (21.15–23.61)	22.15 ± 3.71 (20.90–23.40)	22.51 ± 3.58 (21.29–23.73)	21.87 ± 3.80 (20.59–23.15)	*F* = 0.294, *df* = 3	0.830
Disease Duration (months)	9.12 ± 0.20 (9.06–9.18)	9.14 ± 0.18 (9.08–9.20)	9.13 ± 0.19 (9.07–9.19)	9.16 ± 0.17 (9.11–9.21)	*F* = 0.157, *df* = 3	0.925
Preoperative Hemorrhoid Lesion Area (cm^2^)	12.27 ± 0.32 (12.18–12.36)	12.30 ± 0.34 (12.20–12.40)	12.29 ± 0.31 (12.20–12.38)	12.35 ± 0.33 (12.26–12.44)	*F* = 0.216, *df* = 3	0.885
Preoperative Constipation History [*n* (%)]	12 (30.0%)	11 (27.5%)	13 (32.5%)	10 (25.0%)	*χ*^2^ = 0.685, *df* = 3	0.877

Data are presented as *n* (%) for categorical variables, or mean ± SD (95% CI) for continuous variables. Statistical analyses: *χ*^2^ test for gender; one-way ANOVA for continuous variables. BMI: body mass index.

### 3.2 Primary outcome: postoperative Day 14 wound complete epithelialization rate

The four groups exhibited significant differences in the wound complete epithelialization rate on postoperative day 14 (POD14) [*χ*^2^(3) = 21.64, *P* < 0.001]. Following Bonferroni correction (*α* = 0.0125), the combined group achieved the highest rate (95.0%, 38/40), which was significantly higher than that of the control group [60.0%, 24/40; pairwise *χ*^2^(1) = 12.87, *P* < 0.001], with no significant differences between the combined group and either the fumigation-alone [77.5%, 31/40; pairwise *χ*^2^(1) = 4.52, *P* = 0.033 > 0.0125] or suppository-alone [75.0%, 30/40; pairwise *χ*^2^(1) = 5.71, *P* = 0.017 > 0.0125] groups. Wound complete epithelialization was defined as full surgical wound coverage with intact epithelium and no residual ulceration, confirmed independently by two attending surgeons via anoscopy with excellent inter-rater agreement (Kappa = 0.92, *P* < 0.001). Secondary outcomes were consistent with the primary endpoint: the combined group had the shortest wound healing time (5.15 ± 0.60 days, 95% CI: 4.98–5.32), with significant overall intergroup differences [one-way ANOVA, *F*(3,156) = 38.67, *P* < 0.001]. Blinded anoscopic assessment confirmed these findings, precluding epithelialization status misclassification, and all 160 patients completed POD14 anoscopy with good tolerance, with no examinations interrupted or declined due to postoperative anal pain or edema.

To distinguish additive and synergistic effects of the two interventions, we performed a two-way ANOVA with TCM fumigation and Musk hemorrhoid suppository as binary factors (yes/no). This analysis revealed significant main effects for fumigation alone [*F*(1,156) = 12.36, *P* < 0.001] and suppository alone [*F*(1,156) = 10.72, *P* < 0.001]; more importantly, a significant synergistic interaction effect was identified [*F*(1,156) = 8.59, *P* = 0.004]. Specifically, the 2.30-day reduction in wound healing time with the combined regimen relative to the control exceeded the sum of individual effects of fumigation alone (1.22-day reduction) and suppository alone (1.09-day reduction; 1.22 + 1.09 = 2.31 days), confirming a synergistic therapeutic effect. The combined group also had the highest POD7 wound healing rate (87.5%, 35/40), with significant overall intergroup differences [*χ*^2^(3) = 42.15, *P* < 0.001]; *post-hoc* Bonferroni-corrected comparisons demonstrated this rate was significantly higher than those of the control (32.5%, 13/40; *P* < 0.001), fumigation-alone (65.0%, 26/40; *P* = 0.004), and suppository-alone (62.5%, 25/40; *P* = 0.002) groups. The shorter wound healing time in the combined group was associated with superior granulation tissue quality (0.82 ± 0.31 vs. 1.75 ± 0.43 in the control group; one-way ANOVA, *F* = 29.45, *P* < 0.001) and reduced inflammatory activity via NF-κB/IL-6 pathway inhibition, in addition to standardized wound care protocols.

Further correlational analysis in the combined group revealed granulation tissue quality scores were negatively correlated with VEGF levels (*r* = −0.50, *P* < 0.001) and positively correlated with MMP-9 levels (*r* = −0.50, *P* < 0.001; Supplementary Table S1 and Figure S2), suggesting regulation of VEGF/MMP-9 signaling—marked by decreased VEGF and increased MMP-9 expression in granulation tissue—was associated with improved granulation quality. Consistent with the pathological changes, Western blot analysis revealed a distinct gradient in the expression of phosphorylated NF-κB p65 (p-NF-κB p65, 65kDa) across treatment groups (Supplementary Figure S1). The band intensity was highest in the control group, followed by the fumigation and suppository monotherapy groups, and was significantly lowest in the combined therapy group (0.29 ± 0.08 vs. 0.67 ± 0.11 in the control group, *P* < 0.001). This trend directly verifies the potent inhibitory effect and potential synergism of the combined regimen on the NF-κB signaling pathway. Immunohistochemical analysis further demonstrated that VEGF protein expression in the combined group was 42.3% lower than that in the control group, a reduction calculated using the relative optical density (OD) values of IHC staining via the following formula: (1) Reduction rate (%) = [Mean OD_VEGF (control)−Mean OD_VEGF (combined)]/Mean OD_VEGF (control) × 100% (0.31 ± 0.09 vs. 0.54 ± 0.13, *P* < 0.001), while MMP-9 protein expression was 38.6% higher (0.62 ± 0.12 vs. 0.45 ± 0.10, *P* < 0.001; Supplementary Figure S3).

Notably, the wound healing process was visualized via representative wound images and heat maps (Supplementary Figure S4), which show dynamic surgical wound changes in the combined and control groups at postoperative day 0 (D0, immediate post-operation), D7, D14, and D22, confirming accelerated epithelialization in the combined group. The reliability of wound epithelialization assessments was validated by two blinded attending surgeons and objective metrics (healing time, granulation scores, inflammatory factors), collectively confirming the robustness of our study outcomes.

### 3.3 Secondary outcome: total effective rate

The total effective rate at 4 weeks post-treatment—assessed per The American Society of Colon and Rectal Surgeons Clinical Practice Guidelines for the Management of Hemorrhoids (18)—differed significantly across the four groups (overall *χ*^2^ = 26.847, *df* = 6, *P* < 0.001), with detailed efficacy data presented in Table 2. The combined group achieved the highest total effective rate (97.5%, 39/40), which was significantly higher than that of the control group [65.0%, 26/40; pairwise *χ*^2^(1) = 15.69, *P* < 0.001]. After Bonferroni correction, no significant differences were observed between the combined group and either the fumigation-alone [82.5%, 33/40; pairwise *χ*^2^(1) = 4.50, *P* = 0.034 > 0.0125] or suppository-alone [80.0%, 32/40; pairwise *χ*^2^(1) = 6.05, *P* = 0.014 > 0.0125] groups.

**Table 2:** Comparison of clinical efficacy Among the four groups (4 weeks post-treatment).

Group	Cure [*n* (%)]	Improvement [*n* (%)]	Inefficacy [*n* (%)]	Total effective rate (%)
Control	15 (37.50)	11 (27.50)	14 (35.00)	65.00
Fumigation	22 (55.00)	11 (27.50)	7 (17.50)	82.50
Suppository	21 (52.50)	11 (27.50)	8 (20.00)	80.00
Combined	25 (62.50)	14 (35.00)	1 (2.50)	97.50
Overall	—	—	—	*χ*^2^ = 26.847, *df* = 3, *P* < 0.001
Pairwise comparisons	Combined vs. Control: *P* < 0.001; Combined vs. Fumigation: *P* = 0.008; Combined vs. Suppository: *P* = 0.006; Fumigation vs. Control: *P* = 0.011			

Efficacy criteria: Cure=Resolution of wound redness, swelling, and pain; smooth anal mucosa (confirmed by anoscopy); and normal defecation. Improvement = Reduction in symptoms; presence of fresh granulation tissue (with incomplete epithelialization); and occasional mild discomfort. Inefficacy = No symptom relief; necrotic wound; and presence of exudation. Categorical data are presented as *n* (%). All pairwise comparisons were adjusted using Bonferroni correction (*α* = 0.0125), with *α* = 0.0125 defined as statistically significant. Pairwise comparison results: Combined vs. Control: *P* < 0.001; Combined vs. Fumigation: *P* = 0.008 > 0.0125; Combined vs. Suppository: *P* = 0.006 > 0.0125; Fumigation vs. Control: *P* = 0.011 > 0.0125.

Efficacy tier stratification revealed significant intergroup distribution differences: the combined group had 25 cured cases (62.5%), 14 improved cases (35.0%), and 1 ineffective case (2.5%), compared with 15 cured (37.5%), 11 improved (27.5%), and 14 ineffective (35.0%) cases in the control group. These results align with the primary epithelialization rate endpoint, confirming the intervention's clinical benefit.

### 3.4 Secondary outcome: anal function recovery

All patients successfully completed anorectal manometry on POD14 with no adverse events related to poor examination tolerance. Baseline anal function parameters were comparable across all four groups (all *P* > 0.05). On POD14 (inflammatory resolution phase), all anal function parameters were significantly improved relative to baseline, with the combined group showing the most pronounced improvements; one-way ANOVA confirmed significant intergroup differences in all parameters (all *P* < 0.01, Table 3).

**Table 3:** Comparison of anal function indicators Among the four groups (postoperative Day 14).

Indicator	Control (40)	Fumigation (40)	Suppository (40)	Combined (40)	*F*	*P*
Maximum Anal Squeeze Pressure (mmHg)	113.35 ± 12.18 (109.42–117.28)	116.52 ± 12.54 (112.48–120.56)	115.87 ± 12.36 (111.90–119.84)	121.23 ± 13.26 (116.71–125.75)	5.287	<0.001
Anal Resting Pressure (mmHg)	50.12 ± 4.29 (48.71–51.53)	52.08 ± 4.32 (50.66–53.50)	51.95 ± 4.27 (50.55–53.35)	53.68 ± 4.42 (52.22–55.14)	6.103	<0.001
Anal High-Pressure Zone Length (cm)	3.02 ± 0.37 (2.90–3.14)	2.85 ± 0.29 (2.76–2.94)	2.88 ± 0.31 (2.78–2.98)	2.62 ± 0.23 (2.55–2.69)	12.742	<0.001

Data are presented as mean ± SD (95% CI). Statistical analysis: One-way ANOVA was used for between-group comparisons. All pairwise comparisons were adjusted using Bonferroni correction (*α* = 0.0125), with *α* = 0.0125 defined as statistically significant.

The combined group had an anal resting pressure of 53.68 ± 4.42 mmHg (95% CI: 52.45–54.91; *F* = 6.103, *df* = 3,156, *P* = 0.001), 7.4% higher than the control group (50.12 ± 4.29 mmHg; MD = 3.56 mmHg, 95% CI: 1.24–5.88), with no significant differences from the single-treatment groups (Bonferroni tests, *P* = 0.023–0.031 > 0.0125). The combined group also had a maximum anal squeeze pressure of 121.23 ± 13.26 mmHg (95% CI: 117.15–125.31; *F* = 5.287, *df* = 3,156, *P* = 0.002), 7.0% higher than the control group (113.35 ± 12.18 mmHg; MD = 7.88 mmHg, 95% CI: 2.15–13.61), and an anal high-pressure zone length of 2.62 ± 0.23 cm (95% CI: 2.55–2.69; *F* = 12.742, *df* = 3,156, *P* < 0.001), 13.2% shorter than the control group (3.02 ± 0.37 cm; MD = −0.40 cm, 95% CI: −0.58 to −0.22) and significantly shorter than the single-treatment groups (Bonferroni tests, *P* = 0.003–0.012).

Anal high-pressure zone length reflects the coordinated contraction ability of the internal and external anal sphincters; longer lengths often indicate anal sphincter spasm or dysfunction, which may cause poor fecal continence or increased postoperative pain. A reduction to the near-normal range (2.5–3.0 cm in healthy adults) suggests improved sphincter coordination and restored fecal control (18).

### 3.5 Secondary outcome: symptom improvement

On the 14th day after surgery, symptom severity (assessed via Hemorrhoid Symptom Score [HISS] and Visual Analog Scale [VAS]) in all four groups was significantly relieved compared with pre-treatment (paired *t*-tests for each group, all *P* < 0.001), with the combined treatment group showing the most prominent improvement (Table 4).

**Table 4:** Comparison of symptom scores Among the four groups (postoperative Day 14).

Indicator	Control (40)	Fumigation (40)	Suppository (40)	Combined (40)	*F*	*P*
Pain (VAS Score)	1.72 ± 0.42 (1.57–1.87)	1.53 ± 0.39 (1.40–1.66)	1.49 ± 0.37 (1.37–1.61)	1.27 ± 0.34 (1.16–1.38)	10.862	<0.001
Wound Edema Score	1.58 ± 0.71 (1.35–1.81)	1.25 ± 0.63 (1.05–1.45)	1.28 ± 0.65 (1.08–1.48)	0.89 ± 0.55 (0.72–1.06)	9.743	<0.001
Wound Exudation Score	1.26 ± 0.62 (1.05–1.47)	1.05 ± 0.53 (0.87–1.23)	1.02 ± 0.51 (0.85–1.19)	0.81 ± 0.43 (0.67–0.95)	11.528	<0.001

Data are presented as mean ± SD (95% CI). Statistical analysis: One-way ANOVA. Scoring criteria: VAS (0 = no pain, 10 = severe pain); Edema/Exudation (0 = mild/no symptom, 3 = severe/necrotic [edema] or >12 gauze layers [exudation]). All pairwise comparisons were adjusted using Bonferroni correction (*α* = 0.0125), with *α* = 0.0125 considered statistically significant.

Pre-treatment HISS total scores were comparable across groups (control: 11.2 ± 2.1; fumigation: 10.9 ± 2.3; suppository: 11.1 ± 2.2; combined: 10.8 ± 2.0; *F* = 0.214, *df* = 3,156, *P* = 0.887). After 14 days of treatment, one-way ANOVA showed significant differences in HISS total scores among groups (*F* = 42.67, *df* = 3,156, *P* < 0.001). The combined group had the lowest score (4.2 ± 1.3, 95% CI: 3.7–4.7), which was 46.2% lower than the control group (7.8 ± 1.5, 95% CI: 7.2–8.4; MD = −3.6, 95% CI: −4.3– to −2.9; Bonferroni *post-hoc* test, *P* < 0.001) and significantly lower than the fumigation alone group (5.9 ± 1.4, 95% CI: 5.4–6.4; Bonferroni test, *P* = 0.002) and suppository alone group (6.1 ± 1.6, 95% CI: 5.5–6.7; Bonferroni test, *P* = 0.003) after Bonferroni correction (*α* = 0.0125).

Subgroup analysis of HISS items showed the combined group had the greatest reductions in key symptoms: pain (one-way ANOVA, *F* = 38.24, *P* < 0.001; combined: 1.27 ± 0.34 vs. control: 1.72 ± 0.42, Bonferroni test, *P* < 0.001), bleeding (one-way ANOVA, *F* = 41.56, *P* < 0.001; combined: 0.3 ± 0.4 vs. control: 1.2 ± 0.5, Bonferroni test, *P* < 0.001), and prolapse (one-way ANOVA, *F* = 35.89, *P* < 0.001; combined: 0.5 ± 0.5 vs. control: 1.5 ± 0.6, Bonferroni test, *P* < 0.001)—consistent with the overall HISS total score trend and confirming the combined regimen's multi-symptom relief effect.

### 3.6 Secondary outcome: inflammatory response

Inflammatory factor analysis showed the combined intervention significantly inhibited the NF-κB/IL-6 pathway at both transcriptional and systemic levels, consistent with symptom improvement and wound healing trends (Table 5).

**Table 5:** Comparison of inflammatory indicators Among the four groups (postoperative Day 14).

Indicator	Unit	Control (40)	Fumigation (40)	Suppository (40)	Combined (40)	*F*	*P*
Serum IL-6	pg/mL	7.20 ± 3.10 (6.15–8.25)	5.92 ± 2.85 (4.98–6.86)	6.05 ± 2.91 (5.10–7.00)	4.83 ± 2.76 (3.92–5.74)	8.742	<0.001
Serum TNF-α	pg/mL	10.39 ± 3.26 (9.28–11.50)	8.86 ± 2.94 (7.87–9.85)	8.95 ± 3.02 (7.94–9.96)	7.22 ± 2.37 (6.44–8.00)	7.516	<0.001
Serum CRP	mg/L	9.26 ± 3.34 (8.09–10.43)	7.85 ± 2.98 (6.87–8.83)	7.92 ± 3.05 (6.92–8.92)	6.38 ± 2.65 (5.57–7.19)	6.934	<0.001
Wound NF-κB p65	Relative expression	0.68 ± 0.14 (0.63–0.73)	0.51 ± 0.12 (0.47–0.55)	0.53 ± 0.13 (0.49–0.57)	0.33 ± 0.11 (0.29–0.37)	42.156	<0.001
Wound IκBα	Relative expression	0.98 ± 0.30 (0.88–1.08)	1.35 ± 0.38 (1.22–1.48)	1.32 ± 0.36 (1.20–1.44)	1.81 ± 0.44 (1.67–1.95)	31.829	<0.001
Wound IL-6 mRNA	Relative fold change	4.29 ± 1.05 (3.94–4.64)	3.25 ± 0.89 (2.97–3.53)	3.38 ± 0.92 (3.09–3.67)	2.18 ± 0.68 (1.96–2.40)	38.752	<0.001

Data are presented as mean ± SD (95% CI). Statistical analysis: One-way ANOVA. All pairwise comparisons were adjusted using Bonferroni correction (*α* = 0.0125), with *α* = 0.0125 defined as statistically significant.

On postoperative day 14, one-way ANOVA confirmed significant group differences in wound tissue mRNA markers and serum inflammatory factors (all *P* < 0.001). The combined group had lower pro-inflammatory mRNA levels than the control: IL-6 (2.18 ± 0.68 vs. 4.29 ± 1.05, relative fold change), TNF-α (1.95 ± 0.52 vs. 3.82 ± 0.87), and NF-κB p65 (1.76 ± 0.41 vs. 3.51 ± 0.72) (all Bonferroni, *P* < 0.001), and lower than single-treatment groups (*P* < 0.01). Its anti-inflammatory IκB*α* mRNA was 84.7% higher (1.81 ± 0.44 vs. control 0.98 ± 0.30, *P* < 0.001).

Serum inflammatory factors were measured by ELISA, and the combined group showed significantly reduced levels compared with the control: IL-6 (33.0% lower, 4.83 ± 2.76 vs. 7.20 ± 3.10 pg/mL), TNF-α (30.6% lower, 7.22 ± 2.37 vs. 10.39 ± 3.26 pg/mL), and CRP (31.1% lower, 6.38 ± 2.65 vs. 9.26 ± 3.34 mg/L) (all *P* < 0.001), with single-modality groups showing intermediate reductions (*P* < 0.0125, Bonferroni). Individual data for all inflammatory cytokines and related mRNA markers are presented as scatter plots in Supplementary Figure S5, confirming consistent intergroup differences. Correlation analysis further revealed that NF-κB p65 protein

levels were positively associated with both wound healing time (*r* = 0.62, *P* < 0.001) and HISS score (*r* = 0.58, *P* < 0.001), directly linking NF-κB pathway inhibition to improved clinical outcomes.

### 3.7 Secondary outcome: complications and quality of life

Overall, complication rates, wound healing time, adverse event (AE) incidence, and quality of life scores differed significantly among the four groups. Detailed data are presented in Table 6.

**Table 6:** Postoperative complications and quality of life scores in each group.

Indicator	Control (40)	Fumigation (40)	Suppository (40)	Combined (40)	Statistic	*P*
Complications						
Total incidence [% (n)]	22.50 (9/40)	15.00 (6/40)	17.50 (7/40)	7.50 (3/40)	*χ*^2^ = 8.36, *df* = 3	0.032
Grade I [% (n)]	15.00 (6/40)	10.00 (4/40)	12.50 (5/40)	5.00 (2/40)	—	—
Grade II [% (n)]	7.50 (3/40)	5.00 (2/40)	5.00 (2/40)	2.50 (1/40)	—	—
Wound Healing Time (days)	7.45 ± 0.75 (7.21–7.69)	6.23 ± 0.67 (6.03–6.43)	6.36 ± 0.70 (6.15–6.57)	5.15 ± 0.60 (4.98–5.32)	*F* = 38.67, *df* = 3	<0.001
Total AE Incidence [% (n)]	7.50 (3/40)	2.50 (1/40)	5.00 (2/40)	5.00 (2/40)	*χ*^2^ = 2.15, *df* = 3	0.542
GQOL-30 Scores						
Total score	59.52 ± 3.61 (58.35–60.69)	60.83 ± 3.82 (59.58–62.08)	60.55 ± 3.76 (59.32–61.78)	62.87 ± 4.15 (61.52–64.22)	*F* = 12.54, *df* = 3	<0.001
Physiological function	16.19 ± 1.74 (15.62–16.76)	16.72 ± 1.81 (16.13–17.31)	16.65 ± 1.78 (16.07–17.23)	17.48 ± 1.89 (16.86–18.10)	*F* = 9.82, *df* = 3	<0.001
Social function	13.25 ± 1.64 (12.71–13.79)	13.86 ± 1.69 (13.30–14.42)	13.72 ± 1.67 (13.17–14.27)	14.38 ± 1.72 (13.81–14.95)	*F* = 5.217, *df* = 3	0.002
Psychological function	15.02 ± 1.93 (14.41–15.63)	15.78 ± 1.98 (15.15–16.41)	15.63 ± 1.95 (15.01–16.25)	16.54 ± 2.03 (15.88–17.20)	*F* = 6.943, *df* = 3	<0.001
Material life	14.06 ± 2.08 (13.37–14.75)	14.47 ± 2.12 (13.76–15.18)	14.35 ± 2.10 (13.64–15.06)	15.02 ± 2.15 (14.29–15.75)	*F* = 2.874, *df* = 3	0.036
Granulation Score	1.75 ± 0.43 (1.60–1.90)	1.18 ± 0.39 (1.05–1.31)	1.22 ± 0.41 (1.08–1.36)	0.82 ± 0.31 (0.72–0.92)	*F* = 29.45, *df* = 3	<0.001

Complications were graded using the Clavien-Dindo classification (Grade I: no intervention required; Grade II: requiring pharmacological intervention). GQOL-30: Generic Quality of Life Inventory-30. Continuous data are presented as mean ± SD (95% CI) and analyzed using one-way ANOVA with Bonferroni correction (*α* = 0.0125). Categorical data (complications, adverse events) were analyzed using the *χ*^2^ test, with pairwise comparisons adjusted using Bonferroni correction (*α* = 0.0125). Granulation score criteria: 0 = bright red, firm, and well-vascularized; 1 = pale red with mild edema; 2 = slough-covered with moderate exudation; 3 = necrotic/erosive with heavy exudation. All pairwise comparisons were adjusted using Bonferroni correction, with *α* = 0.0125 defined as statistically significant.

Complication rates varied significantly across groups [*χ*^2^(3) = 8.36, *P* = 0.032]. The combined group had the lowest rate (7.50%, 3/40): 2 Grade I anal distension (no treatment) and 1 Grade II mild exudation (local dressing). The complication rate of the combined group was lower than that of the control group (22.50%, 9/40: 6 Grade I skin irritation, 2 Grade II perianal edema, 1 Grade II minor bleeding; pairwise *χ*^2^ = 4.52, *df* = 1, *P* = 0.033 > 0.0125, no statistically significant difference after Bonferroni correction). No Grade Ⅲ complications occurred.

The combined group had the highest Generic Quality of Life Inventory-30 (GQOL-30) total score (62.87 ± 4.15, 95% CI: 61.52–64.22; ANOVA, *F* = 12.54, *df* = 3,156, *P* < 0.001), significantly higher than the control (59.52 ± 3.61), fumigation (60.83 ± 3.82), and suppository (60.55 ± 3.76) groups (Bonferroni, all *P* < 0.01). The greatest improvement was in physiological function (17.48 ± 1.89 vs. control 16.19 ± 1.74; ANOVA, *F* = 9.82, *P* < 0.001), followed by social (14.38 ± 1.72) and psychological (16.54 ± 2.03) functions.

### 3.8 Validation cohort results

To verify result stability, an internal validation cohort (40 patients, baseline balanced with main cohort, all *P* > 0.05) received the combined intervention, with results consistent with the main cohort: total effective rate 95.00% (38/40; 23 cured, 15 improved, 2 ineffective), not different from main combined group's 97.50% (Fisher's exact test, *P* = 0.618); wound healing time 5.18 ± 0.63 days (95% CI: 4.99–5.37), 2.27 days shorter than concurrent control (7.45 ± 0.72 days, 30.5% reduction vs. main cohort's 30.8%; *t*-test, *t* = 11.28, *df* = 78, *P* < 0.001); inflammatory molecules: wound NF-κB p65 (0.34 ± 0.12) 50.9% lower than control's 0.69 ± 0.15 (*t* = 10.36, *P* < 0.001), IL-6 mRNA (2.23 ± 0.71) 48.6% lower than control's 4.34 ± 1.08 (*t* = 9.82, *P* < 0.001), matching main cohort trends.

### 3.9 Safety

No severe adverse events (AEs) leading to study withdrawal occurred in any group; mild AEs were manageable: the control group had 3 cases (7.50%) of perianal skin irritation (erythema/pruritus, relieved by adjusting potassium permanganate to 1:6,000); the fumigation group had 1 case (2.50%) of transient local burning (spontaneously resolved); the suppository group had 2 cases (5.00%) of mild anal foreign body sensation (adapted to after 1–2 days); the combined group had 2 cases (5.00%) of anal distension (resolved by adjusting suppository insertion depth to 2–3 cm from the anal margin).

Liver function (ALT, AST) and kidney function (Cr) remained within normal ranges pre- and post-treatment in all groups (paired t-tests for pre-post comparisons, all *P* > 0.05), with no drug-related organ damage. Overall chi-square test showed no significant difference in AE incidence among groups (*χ*^2^ = 2.15, *df* = 3, *P* = 0.542) (Table 6).

### 3.10 Subgroup analysis

Subgroup analyses were performed by mixed hemorrhoid subtypes (varicose, circular, connective tissue) to explore efficacy differences. For all subtypes, the combined group had higher total effective rates (varicose: 97.14%, circular: 95.00%, connective tissue: 98.00%) than the control group (62.86%, 60.00%, 66.67%, respectively). Statistical analyses included *χ*^2^ tests for categorical outcomes (total effective rate) and one-way ANOVA for continuous outcomes (serum IL-6 levels), both with Bonferroni correction (*α* = 0.0125); all *P* values for total effective rate comparisons were <0.0125. Additionally, the combined group exhibited more pronounced serum IL-6 reductions (32.5%–34.2%) vs. minimal reductions in the control group (0%–2.1%, one-way ANOVA with Bonferroni correction, all *P* < 0.001). Notably, in the severe circular subtype (*n* = 12), the combined group's wound healing time (5.82 ± 0.65 days) was longer than that of other subtypes in the same group (4.98 ± 0.52 days; t-test, *P* = 0.021) but remained shorter than that of the severe circular subtype in the control group (7.95 ± 0.82 days; *t* = 7.35, *df* = 22, *P* < 0.001).

## 4 Discussion

MMH remains the gold standard for severe mixed hemorrhoids, but postoperative pain and associated postoperative inflammation remain unresolved challenges in Western clinical practice—with recent mechanistic research highlighting targets like the P2X7/ERK axis in dorsal root ganglia (19). Pain drives dual harm: it correlates with heightened inflammation (a key mediator of delayed wound healing) (20, 21) and delays wound repair, underscoring the need for evidence-based, multi-target interventions consistent with Western priorities for reproducibility and objective endpoints (21).

Western postoperative care (e.g., 1:5,000 potassium permanganate sitz baths) has limitations: as noted in European guidelines on wound antiseptics, such regimens primarily exert bactericidal effects [consistent with reported efficacy in reducing wound colonization in anorectal conditions (22)] but lack targeted action on inflammatory pathways or tissue repair mechanisms. Additionally, adjunctive antibiotics for postoperative infection prevention carry inherent risks of resistance, aligning with concerns raised in antiseptic application consensus (22). TCM interventions offer multi-target benefits: herbal fumigation, a modality supported by clinical evidence in anal fistula postoperative care—showing transdermal delivery of active components to regulate local blood flow and mitigate inflammation (1)—delivers synergistic effects on circulation and inflammation. Meanwhile, musk suppositories (containing musk ketone and borneol) alleviate pain and accelerate healing through pathways like COX-2/5-LOX inhibition, consistent with TCM's broader role in enhancing wound repair after anorectal surgery (16).

Our RCT demonstrated that the combined TCM group yielded favorable clinical outcomes relative to the control group, with numerically better results than the two single-modal groups: it achieved a 97.5% total effective rate (39/40), vs. 65.00% in the control group (*P* < 0.001), 82.50% in the fumigation-alone group (*P* = 0.034 > 0.0125), and 80.00% in the suppository-alone group (*P* = 0.014 > 0.0125). Notably, no statistically significant differences emerged between the combined group and either single-modal group following Bonferroni correction (*α* = 0.0125), and this correction was consistently applied to all secondary outcomes and subgroup analyses to minimize false positive risks, with consistent findings in the internal validation cohort (95.00% total effective rate, *P* = 0.618 vs. the main combined group). Furthermore, the combined group achieved the shortest wound healing time (5.15 ± 0.60 days), representing a 2.30-day (30.8%) reduction compared with the control group (7.45 ± 0.75 days, *P* < 0.001).

Notably, our findings on wound healing time align with contemporary evidence: Medina-Gallardo et al. (8) (2022, *Dis Colon Rectum*) reported that targeted anti-inflammatory intervention (local bupivacaine + triamcinolone infiltration) reduced MMH postoperative healing time to a mean of 8.0 ± 1.2 days; a study (19) further confirmed that anti-inflammatory-focused interventions shortened MMH healing to 6.5 ± 0.8 days. In contrast, 87.5% of our combined group healed within 7 days, attributed to superior granulation quality (score: 0.82 ± 0.31 vs. the control group's 1.75 ± 0.43; *F* = 29.45, *P* < 0.001) and reduced NF-κB p65 expression (51.5% lower vs. the control group)—thereby minimizing inflammatory delays in wound healing.

By day 14, the combined group exhibited 35.7% less exudation, a 26.2% reduction in pain VAS scores, and a 66.7% relative reduction in complication rate (7.50% vs. 22.50% in the control group; nominal *χ*^2^ = 8.36, *P* = 0.032, non-significant after Bonferroni correction), with no severe adverse events reported. Additionally, the group showed improved anal function: resting pressure increased by 7.4%, maximum squeeze pressure by 7.0%, and anal high-pressure zone length decreased by 13.2% (all *P* < 0.001), alongside significantly higher GQOL-30 total scores (62.87 ± 4.15 vs. 59.52 ± 3.61 in the control group; *F* = 12.54, *P* < 0.001) with the most notable improvement in the physiological function dimension.

Notably, the combined group only demonstrated statistically significant superiority over the control group (all *P* < 0.001 after Bonferroni correction), while differences between the combined group and the single-modal groups were not statistically significant—highlighting that the observed interactive effect is most evident when compared to conventional standard care. The combined regimen's efficacy is associated with suppression of the NF-κB/IL-6 pathway: on postoperative day 14, it reduced mRNA levels of pro-inflammatory cytokines (IL-6, TNF-α) and NF-κB p65 (a key transcription factor in the pathway), while upregulating anti-inflammatory IκB*α* mRNA in wound tissue (all *P* < 0.001); it also lowered serum pro-inflammatory cytokines (IL-6, TNF-α, CRP) by 30.6%–33.0% vs. the control group (*P* < 0.001). These findings confirm that NF-κB/IL-6 pathway suppression occurs at both the transcriptional and systemic levels. The quantified concentrations of chlorogenic acid, matrine, and phillyrin in the fumigation filtrate and their perianal exposure concentrations synergistically mediated this pathway modulation: phillyrin (Forsythia suspensa) suppresses NF-κB activation (13); glycyrrhizin (Glycyrrhiza uralensis) inhibits IL-6 production (14); and matrine (Sophora flavescens) inhibits pathological angiogenesis—a process critical to wound repair—via the HIF-VEGF signaling pathway (15). Notably, NF-κB p65 protein levels (Supplementary Figure S1) correlated significantly with wound healing time (*r* = 0.62, *P* < 0.001) and the Hemorrhoid Symptom Score (HISS, *r* = 0.58, *P* < 0.001), directly linking NF-κB pathway suppression to improved clinical outcomes, with VEGF/MMP-9 modulation further validating this mechanistic link (Supplementary Figure S3).

Subgroup analysis confirmed consistent favorable outcomes across varicose, circular, and connective tissue subtypes of mixed hemorrhoids: effective rates ranged from 97.14% to 98.00% in the combined group vs. 60.00% to 66.67% in the control group (all *P* < 0.05), with IL-6 reductions of 32.5%–34.2% compared with minimal changes (0%–2.1%) in the control group (all *P* < 0.001). In the severe circular mixed hemorrhoid subtype (*n* = 12), healing time in the combined group (5.82 ± 0.65 days) was longer than that of other subtypes within the same group (4.98 ± 0.52 days, *P* = 0.021) but remained significantly shorter than that of the severe circular subtype in the control group (7.95 ± 0.82 days, *P* < 0.001), suggesting a need for subtype-specific adjustments to the intervention.

This study has several limitations: First, the single-center, regionally restricted sample limits external validity and generalizability. Second, the 14-day primary and 4-week secondary endpoints are insufficient to evaluate critical long-term outcomes. This short follow-up lacks data on these key metrics, precluding comprehensive assessment of long-term safety, efficacy, and durability. Third, subgroup analyses are superficial, with no exploration of intervention optimization for subtypes like slow-healing circular hemorrhoids. Fourth, the synergistic mechanism of the 13-herb fumigation formula remains unclear, as only single-component effects were analyzed. Fifth, postoperative defecation habits were overlooked as a confounder, with insufficient data to mitigate potential bias.

## 5 Conclusion

TCM fumigation plus Musk hemorrhoid suppository safely accelerates post-MMH recovery by suppressing inflammation and NF-κB/IL-6 pathway activity—backed by lower pro-inflammatory mRNA/cytokines—while reducing exudation, restoring anal function, and enhancing quality of life. Notably, its efficacy rests on clinical outcomes (30.8% shorter healing time, 66.7% fewer complications) and NF-κB/IL-6 mechanistic evidence, aligning with Western medicine's emphasis on causal pathways and objective data. Standardized procedures boost Western translatability, offering an evidence-based solution for unmet post-MMH needs. It shows consistent subtype efficacy with a favorable safety profile but requires standardized herbal quality control and additional mechanistic research for global use.

## Data Availability

The raw data supporting the conclusions of this article will be made available by the authors, without undue reservation.
