# Supplementary material for: Traditional Chinese medicine fumigation combined with Musk hemorrhoid suppository for wound healing and inflammation control in patients with mixed hemorrhoids after Milligan-Morgan hemorrhoidectomy
Source: Front Surg. 2026 May 20;13:1738476. doi: 10.3389/fsurg.2026.1738476 (PMC13231432; doi:10.3389/fsurg.2026.1738476)
Supplement: Supplementary file 1 [file Supplementaryfile1.docx]

**Supplementary Materials**

**1. Pilot Study Data for Sample Size Calculation**

**1.1 Study Design**

A prospective, single-center, open-label randomized controlled trial conducted at the Third Affiliated Hospital of Liaoning University of Traditional Chinese Medicine (June–December 2022). Stratified randomization was performed according to hemorrhoid grade (grade Ⅲ and Ⅳ) to ensure balanced baseline distribution. The primary objectives were to verify the feasibility of the combined traditional Chinese medicine (TCM) intervention and obtain preliminary efficacy data to inform sample size calculation for the main trial.

**Inclusion/Exclusion Criteria**

Identical to the main trial, inclusion criteria included grade Ⅲ–Ⅳ mixed hemorrhoids confirmed by anoscopy, age 18–65 years, prior uncomplicated Milligan-Morgan hemorrhoidectomy (MMH), no receipt of traditional Chinese medicine (TCM) or immune-modulating therapy within 1 month, and provision of written informed consent, while exclusion criteria encompassed perianal comorbidities (e.g., anal fistula, perianal abscess), severe organ dysfunction, coagulation disorders, inflammatory bowel disease, and cognitive impairment.

**Interventions**

The Combined Group (n=30) received TCM fumigation (a 13-herb formula standardized via High-Performance Liquid Chromatography [HPLC]) combined with Musk hemorrhoid suppositories, administered once daily for 14 days, while the Control Group (n=30) underwent 1:5000 potassium permanganate sitz baths, administered once daily for the same 14-day period.

**Key Outcomes**

The key outcomes included the primary outcome of wound healing time (defined as the number of days from postoperative day 1 to complete epithelialization, confirmed via visual inspection and anoscopy) and secondary outcomes of serum interleukin-6 (IL-6) measured via enzyme-linked immunosorbent assay (ELISA) and wound tissue nuclear factor kappa B (NF-κB) p65 mRNA measured via quantitative real-time polymerase chain reaction (qRT-PCR) (to confirm mechanistic relevance).

**Ethics Approval**

This study was approved by the Medical Ethics Committee of the Third Affiliated Hospital of Liaoning University of Traditional Chinese Medicine (Ethics No.: LLPG-ZY-GC-2022-018). Written informed consent was obtained from all participants prior to enrollment.

**1.2 Baseline Characteristics of the Pilot Study**

No statistically significant between-group differences were observed (all P>0.05), confirming baseline comparability:

| Characteristic | Combined Group (n=30) | Control Group (n=30) | Statistic | P-value |
| --- | --- | --- | --- | --- |
| Age (years, mean±SD) | 39.42±3.31 | 39.55±3.27 | t=0.16 | 0.875 |
| Gender (Male/Female, n%) | 19/11 (63.3%/36.7%) | 20/10 (66.7%/33.3%) | χ2=0.08 | 0.777 |
| Hemorrhoid grade (Ⅲ/Ⅳ, n%) | 17/13 (56.7%/43.3%) | 16/14 (53.3%/46.7%) | χ2 =0.09 | 0.765 |
| Preoperative wound area (cm², mean±SD) | 12.31±0.34 | 12.28±0.32 | t=0.38 | 0.706 |

**1.3 Effect Size Calculation Details**

The primary effect size for sample size calculation was determined based on the primary endpoint of wound healing time from the pilot trial, with detailed calculations and clinical justification provided below:

**1.3.1 Pilot Study Key Results**

The Combined Group had a wound healing time of 5.32±0.65 days, while the Control Group had a wound healing time of 7.68±0.72 days, resulting in a mean difference of 2.36 days (representing a 30.7% relative reduction compared to the Control Group).

**1.3.2 Calculation Steps**

**Pooled SD:**

SD_pooled_ = sqrt[(SD²_control_ + SD²_combined_) / 2] = sqrt[(0.72² + 0.65²) / 2] = 0.686

**Unadjusted Cohen’s d:**

d = (mean_control_ − mean_combined_) / SD_pooled_ = (7.68 − 5.32) / 0.686 ≈ 3.44

**Conservative Effect Size Specification**

Effect sizes derived from small pilot studies are prone to overestimation. To ensure rigorous and conservative sample size estimation for the main trial, the raw large effect size (d=3.44) was not directly adopted. Combined with the reported effect size range of surgical and adjuvant interventions for anorectal diseases (d=2.1-3.8) [1] from published meta-analyses, a conservative effect size of d=2.5 was finally applied for main trial sample size calculation.

**1.3.3 Clinical Justification**

A 2-day reduction in postoperative wound healing time after Milligan–Morgan hemorrhoidectomy addresses an important unmet clinical need. Delayed perianal wound healing is closely associated with increased risks of postoperative pain, infection and other complications, which is well documented in authoritative reviews on hemorrhoid surgical management [2]. The specified effect size (d=2.5) is within the credible range of comparative anorectal intervention studies, reflecting the stable synergistic anti-inflammatory and pro-healing properties of the integrated TCM regimen.

Data Availability

Raw data, statistical analysis code, and quality control reports from the pilot study are available from the corresponding author upon reasonable request.

**Reference**

[1] Cheng PL, Chen CC, Chen JS, Wei PL, Huang YJ. Diode laser hemorrhoidoplasty versus conventional Milligan-Morgan and Ferguson hemorrhoidectomy for symptomatic hemorrhoids: Meta-analysis. Asian J Surg. 2024;47(11):4681-4690. doi:10.1016/j.asjsur.2024.04.156

[2] Altomare DF, Giuratrabocchetta S. Conservative and surgical treatment of haemorrhoids. Nat Rev Gastroenterol Hepatol. 2013;10(9):513-521. doi:10.1038/nrgastro.2013.91

**2 Supplementary Figures**


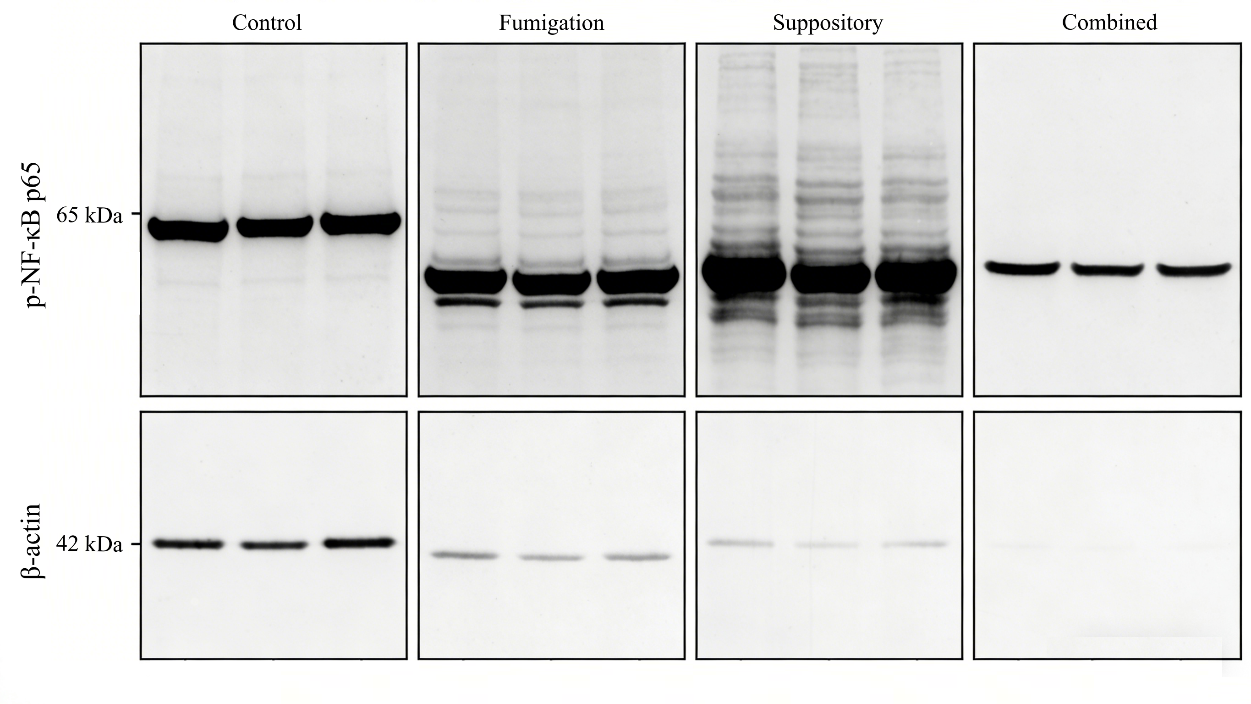


Supplementary Figure S1. Representative Western blot analysis of phosphorylated NF-κB p65 (p-NF-κB p65, 65 kDa) and β-actin (42 kDa, loading control) in wound tissue.

Note: The upper panel shows the expression of p-NF-κB p65, and the lower panel shows the expression of the loading control β-actin. Lanes are labeled as follows: Control (untreated), Fumigation (monotherapy), Suppository (monotherapy), and Combined (combination therapy). A clear reduction in band intensity is observed in the combined therapy group compared to the control and monotherapy groups. The relative expression levels of p-NF-κB p65 were normalized to β-actin levels, which showed consistent expression across all lanes, ensuring valid quantitative analysis.


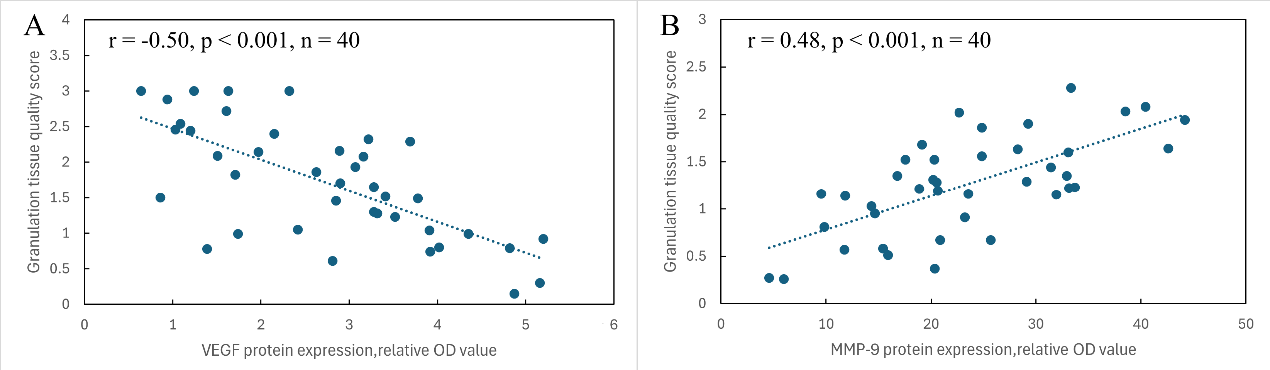


Supplementary Figure S2: Correlation analyses between granulation tissue quality score and angiogenic factor expression in wound healing.

Note: (A) VEGF protein expression (relative OD value) was negatively correlated with the granulation tissue quality score (r = -0.50, p < 0.001, n = 40). (B) MMP-9 protein expression (relative OD value) was positively correlated with the granulation tissue quality score (r = 0.48, p < 0.001, n = 40). Linear regression lines are shown as dotted lines.


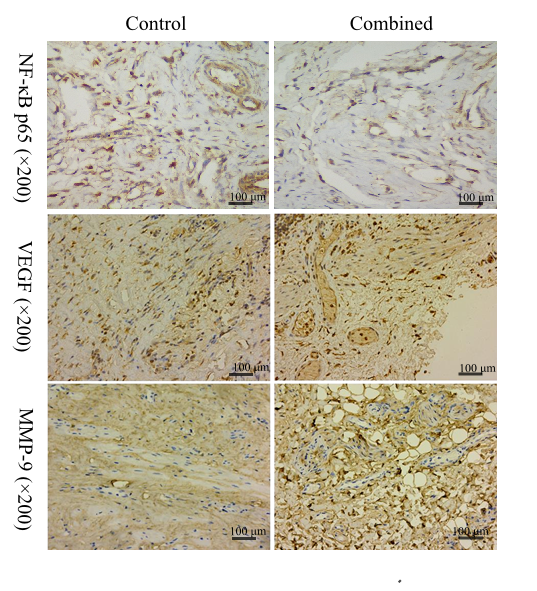


Supplementary Figure S3: Immunohistochemical staining of NF-κB p65, VEGF, and MMP-9 in human anal granulation tissue after mixed hemorrhoid surgery (×200 magnification).

Note: Left column: Control group; Right column: Combined treatment group. Brown staining indicates DAB-positive immunoreactivity, and blue staining indicates hematoxylin counterstained nuclei. Scale bar = 100 μm.


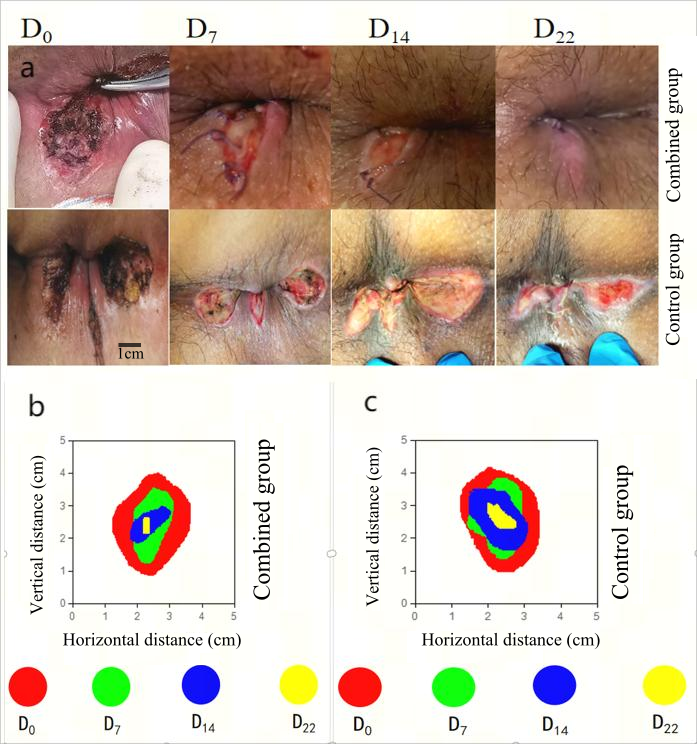


Supplementary Figure S4: Wound healing process in the combined and control groups after Milligan-Morgan hemorrhoidectomy.

Note: (a) Representative wound images at postoperative day 0 (D₀, immediate post-operation), D₇, D₁₄, and D₂₂. Scale bar: 1 cm. (b, c) Wound area heat maps for the combined group and control group, respectively. Red, green, blue, and yellow represent D₀, D₇, D₁₄, and D₂₂, respectively, demonstrating progressive wound shrinkage over time. The combined group exhibited significantly accelerated epithelialization compared with the control group.


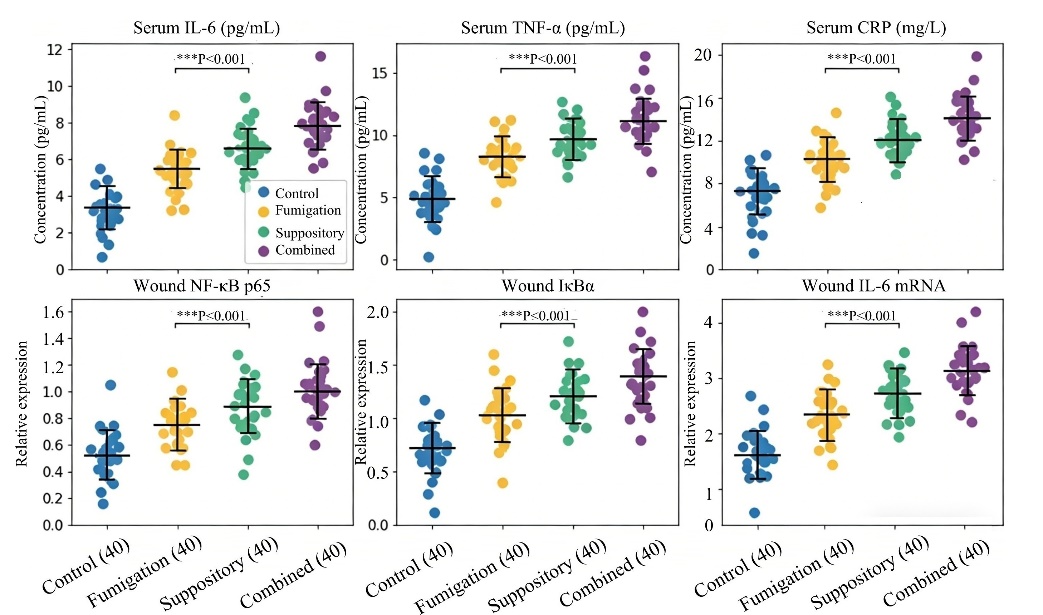


Supplementary Figure S5: Scatter plots of inflammatory cytokines and related indicators in four groups on postoperative day 14.

Note: Each dot represents an individual patient, and horizontal black lines indicate the mean ± standard deviation. Compared with the control group, the combined group showed significantly lower levels of serum IL-6, TNF-α, CRP, wound NF-κB p65, and wound IL-6 mRNA, and a significantly higher level of wound IκBα (***P<0.001; all comparisons via one-way ANOVA with Bonferroni correction).

**3 Supplementary Table**

**Table S1 Correlation between granulation tissue quality score and protein expression levels of VEGF and MMP‑9 in the combined treatment group (n = 40)**

| Correlation index | Pearson correlation coefficient (r) | P value | 95% confidence interval | Correlation trend |
| --- | --- | --- | --- | --- |
| Granulation tissue quality score vs. VEGF protein OD value | −0.50 | <0.001 | −0.69 to −0.31 | Significant negative correlation |
| Granulation tissue quality score vs. MMP‑9 protein OD value | 0.48 | <0.001 | 0.30 to 0.64 | Significant positive correlation |

Note: Pearson correlation analysis was used for data conforming to normal distribution. Granulation tissue quality score was rated from 0 to 3 (0 = best, 3 = worst), and VEGF and MMP‑9 levels were presented as relative OD values from immunohistochemical staining. VEGF, vascular endothelial growth factor; MMP‑9, matrix metallopeptidase 9; OD, optical density.
